# Antibody–Drug Conjugates for Cancer Therapy

**DOI:** 10.3390/molecules25204764

**Published:** 2020-10-16

**Authors:** Umbreen Hafeez, Sagun Parakh, Hui K. Gan, Andrew M. Scott

**Affiliations:** 1 Tumour Targeting Laboratory, Olivia Newton-John Cancer Research Institute, Melbourne, VIC 3084, Australia; umbreen.hafeez@onjcri.org.au (U.H.); sagun.parakh@onjcri.org.au (S.P.); hui.gan@onjcri.org.au (H.K.G.); 2 Department of Medical Oncology, Olivia Newton-John Cancer and Wellness Centre, Austin Health, Melbourne, VIC 3084, Australia; 3 School of Cancer Medicine, La Trobe University, Melbourne, VIC 3084, Australia; 4 Department of Medicine, University of Melbourne, Melbourne, VIC 3084, Australia; 5 Department of Molecular Imaging and Therapy, Austin Health, Melbourne, VIC 3084, Australia

**Keywords:** antibody–drug conjugate, ADC, monoclonal antibody, cytotoxic payload, linkers, cancer, molecular imaging

## Abstract

Antibody–drug conjugates (ADCs) are novel drugs that exploit the specificity of a monoclonal antibody (mAb) to reach target antigens expressed on cancer cells for the delivery of a potent cytotoxic payload. ADCs provide a unique opportunity to deliver drugs to tumor cells while minimizing toxicity to normal tissue, achieving wider therapeutic windows and enhanced pharmacokinetic/pharmacodynamic properties. To date, nine ADCs have been approved by the FDA and more than 80 ADCs are under clinical development worldwide. In this paper, we provide an overview of the biology and chemistry of each component of ADC design. We briefly discuss the clinical experience with approved ADCs and the various pathways involved in ADC resistance. We conclude with perspectives about the future development of the next generations of ADCs, including the role of molecular imaging in drug development.

## 1. Introduction

Despite the range of new anti-cancer drugs in the market, there were 9.6 million deaths from cancer globally in 2018, with approximately one in six of all deaths being due to cancer [1]. A recent review concluded that most of the anti-cancer drugs that entered the market between 2009 and 2013 showed only marginal gains in terms of overall survival and that there is an urgent need to increase therapeutic efficacy [2]. Systemic therapies based on the use of monoclonal antibodies (mAbs) started to emerge after the discovery of hybridoma technology by Kohler and Milstein in 1975. In 1988, Greg Winter pioneered the technique to humanize monoclonal antibodies and thereafter, therapeutic monoclonal antibodies have been successfully developed for the treatment of various cancers. To date, approximately 30 mAbs have been approved by the US Food and Drug Administration (FDA) for the treatment of cancer. The specificity of mAbs also makes them well suited for the targeted delivery of drugs to cancer cells whilst avoiding normal tissues. Monoclonal antibody technology has been further improved over the next decades by conjugating antibodies with cytotoxic drugs [3]. Such conjugates, called antibody–drug conjugates (ADCs), are targeted agents that link a cytotoxic drug (also called as cytotoxic payload or warhead) via a linker to a monoclonal antibody which specifically recognizes a cellular surface antigen and deliver toxic payload at the tumor site, thus improving the efficacy of chemotherapy and reducing systemic exposure and toxicity [4]. More than 80 ADCs are currently under clinical development worldwide [5,6]. In this paper, we provide an overview of the biology and chemistry of ADCs, discuss key components of ADC design and discuss the current status of clinically approved and underdeveloped ADCs. We also discuss resistance mechanisms to ADC, the role of molecular imaging in the clinical development of ADCs and give an opinion on future perspectives.

## 2. Design and Structure of Antibody–Drug Conjugates

**Figure 1:**
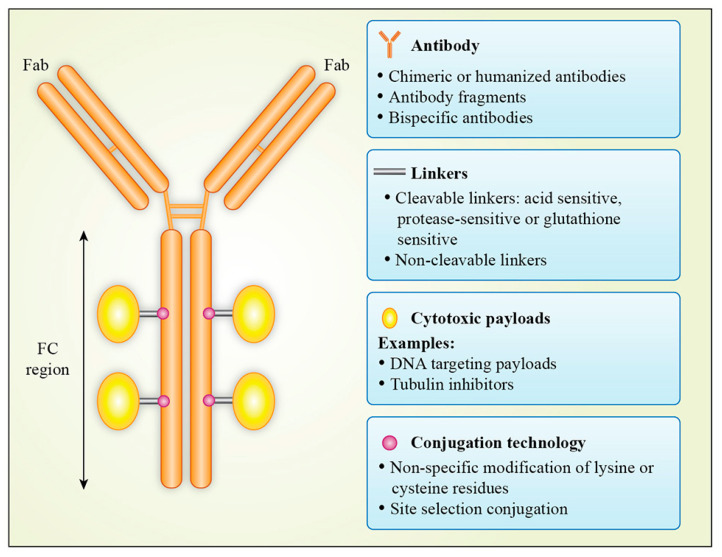
Antibody–drug conjugate structure.

The use of ADCs has evolved over time, becoming increasingly widespread as initial problems were overcome by better target selection, accompanied by improvements in payloads, linker technologies and conjugation techniques (Figure 1).

### 2.1. Target Selection

Unconjugated mAbs have various mechanisms of action including target receptor neutralization, receptor downregulation, signalling disruption, antibody dependent cell-mediated cytotoxicity (ADCC), complement-dependent cell-mediated cytotoxicity (CDCC) and immune checkpoint inhibition [7,8]. On the other hand, ADCs rely on target receptor internalization to deliver the cytotoxic payload to the cancer cells. Hence, selecting an appropriate target antigen is a critical step for the success of an antibody–drug conjugate.

Ideally, the target antigen should be abundant on the cell surface to be available for binding by the circulating ADC, e.g., melanoma cell lines with p97 receptor expression levels from 80,000 to 280,000 receptors per cell showed sensitivity to ADC L49-vcMMAF, while other cancer cell lines with lower p97 expression were resistant to L49-vcMMAF [9]. However, various other ADCs have shown efficacy over a wide range of antigen expression levels based on other characteriztics of the target antigen, including binding affinity and rate of internalization. Gemtuzumab ozogamicin has demonstrated efficacy at a relatively low expression of CD33 (5000 to 10,000 receptors per cell) as compared to trastuzumab emtansine (T-DM1) which usually requires high ErbB2 expression levels (>2 million receptors per cell) [10,11]. The high surface expression of the antigen may not lead to ADC efficacy if trafficking into the tumor cell is impaired. Inge et al. [12] showed that after antibody binding CD21 does not effectively internalize, even when expressed at very high levels, resulting in a lack of efficacy with anti-CD21-MCC-DM1 ADCs. This study also showed that CD21 forms a complex with CD19 on the surface of B cells and prevents internalization of CD19 after it is bound to anti-CD19 antibody and decreasing anti-CD19-MCC-DM1 efficacy [12].

To minimize off-target toxicity, the target antigen should have exclusive or preferential expression on cancer cells with a minimal expression on healthy tissue. Four out of nine approved ADCs—inotuzumab ozogamicin, gemtuzumab ozogamicin, brentuximab vedotin, and polatuzumab vedotin—target lineage-specific markers that include CD22, CD33, CD30 and CD79, respectively. These antigens have consistent expression across the target cell population. The target antigen should also have minimal secretion in circulation from the cancer cells, as antibodies can bind to these secreted receptors in the circulation, thus limiting the amount of antibody available for target cell binding [13].

The target antigen should internalize efficiently upon ADC binding to allow the antigen–ADC complex to be internalized via receptor-mediated endocytosis, and it should undergo appropriate intracellular trafficking and degradation, thereby allowing the cytotoxic warhead to be released [14]. Target antigens on the cell surface can have variable rates for basal and antibody induced internalization which can affect ADC efficacy [12]. For example, CD74 antigen is rapidly internalized into target cells following antibody binding. The preclinical data for anti-CD74 ADC immu-110 showed similar efficacy to other ADCs that target slower internalizing antigens with more potent cytotoxic payloads [15]. Inadequate or inefficient internalization increases the chance of toxicity from inappropriate payload delivery outside the cancer cell [16]. Other factors which influence antigen–ADC internalization are binding epitope on target antigen and the affinity of the ADC to the antigen [10].

Lastly, antigens that are abundant in the circulation should also be avoided to prevent sequestration and/or degradation of the ADC in the circulation. More than 50 different known antigens have been used as targets in ADCs currently in preclinical or clinical development (Table 1).

### 2.2. Choice of Antibody

The selection of an appropriate antibody for ADC is also important and can have a significant impact on efficacy, pharmacokinetic/pharmacodynamic profiles and therapeutic index. An ideal mAb for an ADC should be target specific with strong target binding affinity. In addition, it should have low immunogenicity, low cross-reactivity, efficient internalization and long plasma half-life [17,18]. Antibodies in human beings can be classified into five main classes: IgA, IgD, IgE, IgG, and IgM. These closely related immunoglobulins are composed of 82–96% protein and 4–18% carbohydrate; they differ in heavy chain structure and have distinct effector function. IgA is further subclassified into IgA1 and IgA2. IgG has been subdivided into IgG1, IgG2, IgG3 and IgG4. Due to its favourable characteriztics, scientists have focused on IgG class for therapeutics. An IgG antibody consists of two heavy chains and two light chains. It has two antigen binding fragments (Fabs) which mediate antigen recognition and a constant fragment (Fc), which mediates the binding of the antibody with effector cells of the immune system. The Fc fragment also regulates the half-life of the antibody in circulation through its interaction with FcRn (the neonatal Fc receptor for IgG) [19,20]. The IgG1 subtype is frequently used in antibody therapeutics due it its serum stability and strong binding affinity for Fc receptor. However, an increasing number of ADCs are based on Fc-silent mAbs and the suitability of Fc for an ADC is determined on a case by case basis [8,19,21,22,23,24]. However, Fcγ binding can also lead to undesirable adverse events. T-DM1 binds to FcγRIIa and internalized by megakaryocytes resulting in thrombocytopenia [25]. Another ADC, LOP628 consisting of c-Kit IgG1 antibody conjugated to maytansine, was found to induce hypersensitivity reactions in patients in a phase I trial after mast cell degranulation due to FcγR1 and FcγRII binding on mast cells [26]. Further development of LOP628 ADC has been discontinued. IgG2 and IgG4 have reduced capacity to activate the complement cascade. TR 1801-ADC, an ADC where a c-Met IgG2 antibody is used as a backbone, is currently in phase I clinical trial (Table 3). IgG4 is preferred for the development of ADCs when recruitment of host effector function is least required. However, despite a lower affinity for FcγR compared with IgG1, in its non-fucosylated form, IgG4 antibody is able to bind to FcγRIIIa [25]. IgG4 antibodies have been used in the development of gemtuzumab ozogamicin and inotuzumab ozogamicin (Table 2).

Early ADCs utilized murine antibodies which were highly immunogenic, resulting in reduced efficacy and toxicity due to the development of human anti-murine antibodies (HAMAs) [27,28]. To overcome this problem, chimeric, humanized or fully human antibodies were used in next-generation ADCs. A chimeric antibody contains the mouse antigen binding fragments (Fabs) linked to a human constant fragment (Fc). The human constant fragment mediates interaction with cells of the immune system, and hence immunogenicity is reduced. ADCs that contains chimeric antibodies are brentuximab vedotin and indatuximab ravtansine (Table 2 and Table 3). However, human anti-chimeric antibodies (HACA) could still develop in these patients. In contrast, a humanized antibody contains only the complementarity determining regions of Fab that are grafted into the human Fab regions and hence the risk of human anti-human antibodies (HAHA) is reduced even further. An example of humanized antibody in ADC trastuzumab emtansine is mAb trastuzumab, linked to cytotoxic payload DM1 [29]. The benefit of using a fully human antibody is to prevent mounting an immune response against these antibodies. Enfortumab vedotin is an ADC consisting of fully human anti-nectin-4 mAb which was recently approved by the FDA for management of patients with advanced urothelial cancer (Table 2).

### 2.3. Cytotoxic Payload

The first ADCs utilized readily available conventional cytotoxics such as doxorubicin (e.g., BR96-doxorubicin), methotrexate, mitomycin, fluorouracil and vinca alkaloids [30,31,32,33]. These chemotherapeutic drugs have relatively low potency with half maximal inhibitory concentration (IC_50_) values in the micromolar range, they lacked selectivity and their accumulation in target cells was poor. As a result, this contributed to the relatively low efficacy of these early ADCs [34,35].

Subsequently, more potent payloads have been utilized with IC_50_ values in the subnanomolar range. In addition to potency, plasma stability, low immunogenicity, small molecular weight and a long half-life are also desirable characteriztics for ADC payloads [36]. It was also important that the chemistry of payload should not disrupt the internalization properties of the parental mAb. The most commonly used payloads currently mainly target either DNA or tubulins.

DNA targeting payloads include calicheamicins, duocarmycins, pyrrolobenzodiazepines (PBDs), SN-38 and DXd. Calicheamicins in ADCs inotuzumab ozogamicin and gemtuzumab ozogamicin bind to a minor groove of DNA and induce double-stranded breaks and subsequent cell apoptosis [37]. As duocarmycin is rapidly degraded in plasma, it is incorporated into ADCs in its inactive pro-drug form, seco-duocarmycin-hydroxybenzamide-azaindole (seco-DUBA). Once released inside cells, duocarmycin is attached to the minor groove of DNA in A-T-rich region and causes DNA alkylation, resulting in DNA damage in both dividing and non-dividing cells and ultimately cell death. Duocarmycin is the cytotoxic payload in trastuzumab duocarmazine currently in phase II/III clinical trials [38,39]. The pyrrole[2,1-c] [1,4] benzodiazepines (PBDs) cause DNA alkylation. The naturally occurring PBDs produced by actinomycetes *Streptomyces* and *Micrococcus* (e.g., anthramycin) form single alkylated DNA adducts by binding to guanine residues in the DNA minor groove. However, the synthetic PBD dimers consisting of two PBD units joined through a linker can also form interstrand or intrastrand DNA cross-links. PBD dimers are sequence selective, and are distinguished from conventional DNA cross-linking agents (e.g., platinum drugs), in that the interstrand cross-links formed between DNA and PBD dimer do not cause distortion of DNA and hence go undetected by DNA repair mechanisms, maintaining its activity and ultimately leading to cell death [40,41,42]. Loncastuximab tesirine is an ADC containing anti-CD19 antibody conjugated to a PBD dimer SG3199, and is currently in a phase III clinical trial (Table 3) [42]. SN-38 is an active metabolite of irinotecan. Irinotecan is a topoisomerase I inhibitor that interrupts DNA replication with subsequent cell cycle arrest and cell death. SN-38 is the cytotoxic payload in ADC sacituzumab govitecan [43]. DXd, an exatecan derivative (DX-8951 derivative) is also a topoisomerase I inhibitor. It is ten times more potent than SN-38 [44]. It is used as a payload in a trastuzumab deruxtecan which was recently approved by the FDA for treatment of metastatic breast cancer [45].

The anti-tubulin agents include auristatins and maytansinoids. Auristatins are synthetic analogues of natural agent dolastatin 10, which was first isolated from sea hare *Dolabella Auricularia* in 1987 [46]. They disrupt microtubules and induce cell cycle arrest in the G2/M phase. Four out of nine approved ADCs use auristatins as payloads. Monomethyl auristatin E (MMAE) is used as a warhead in approved ADCs brentuximab vedotin, polatuzumab vedotin and enfortumab vedotin while monomethyl auristatin F (MMAF) was used in depatuxizumab mafodotin. Further development of depatuxizumab mafodotin has been ceased owing to disappointing results in a phase III trial [47,48,49,50]. Maytansinoids are derived from the naturally occurring maytansine, which was originally isolated from the bark of the shrub *Maytenus ovatus* [51]. Maytansine binds tubulin at the vinca-binding site, inhibit depolymerization and induce mitotic arrest [52]. The synthetic maytansinoid derivative mertansine (DM1) is the payload in approved ADCs trastuzumab emtansine and brentuximab vedotin while DM4 is conjugated to coltuximab ravtansine. Coltuximab ravtansine is in phase I/II clinical trials. They all have shown potent cytotoxic activity [53,54,55].

### 2.4. Linkers

The linkers play a significant role in antibody–drug conjugate design as they link the cytotoxic payload to the antibody. Various factors such as linker chemistry, mode and the site of conjugation play a crucial role in the pharmacokinetic and pharmacodynamic properties of ADC. Linkers must maintain the stability of the ADC in the blood circulation to enable it to reach the cancer cell intact but must then be readily cleaved when internalized so that the payload can be released. Broadly speaking, linkers can be categorized into two groups: cleavable or non-cleavable linkers.

Cleavable linkers depend on physiological conditions in the cell to cleave the linker and can be further subdivided into acid sensitive, protease sensitive or glutathione sensitive. The acid-sensitive linker AcBut (4-4′-acetylphenoxy butanoic acid) is used to link anti-CD33 and anti-CD22 antibodies to calicheamicin in ADCs gemtuzumab ozogamicin and inotuzumab ozogamicin, respectively. This linker connecting the antibody to calicheamicin forms a hydrazone with the hydrazide of the calicheamicin derivative, which is hydrolysed in lysosomal pH, and calicheamicin is released, leading to cell death. However, the acid-sensitive linker has a low plasma stability of 48–72 h [56,57,58]. The protease-cleavable linker, e.g., dipeptide valine-citrulline (Val-Cit, V-C), is utilized in brentuximab vedotin to link the monomethylauristatin E (MMAE) payload to the anti-CD30 antibody. Val-Cit is more stable in the plasma due to the presence of protease inhibitors than acid-based linkers, but can still be rapidly hydrolysed by lysosomal enzyme cathepsin B once internalized [55]. Another protease-cleavable valine-alanine (Val-Ala) linker has been used for multiple ADCs, including TR1801 where PBD is conjugated to an anti-cMET antibody [59]. Lastly, glutathione-sensitive disulfide linkers are commonly used in ADC design. Glutathione is released during cell survival and tumor growth. A high concentration of glutathione is found in the intracellular compartment in cancer cells as compared to normal cells [60]. Therefore, glutathione-sensitive linkers are stable in blood circulation and are cleaved by elevated glutathione in the tumor cells, releasing the cytotoxic payload. The ADC mirvetuximab soravtansine (IMGN 853) is comprised of FRα binding mAb M9346A via a glutathione-sensitive cleavable disulfide SPDB linker attached to cytotoxic payload DM4 [61].

The second major category of linkers is non-cleavable linkers. Non-cleavable linkers form non-reducible bonds with the amino acid residues of the mAb and should therefore be more stable in the bloodstream, have longer half-lives and reduced off target toxicity. ADCs involving non-cleavable thioether linkers are efficiently internalized in cells and then degraded in lysosomes to yield metabolites consisting of intact cytotoxic payload and the linker attached to amino acid lysine [62]. T-DM1 has been successfully created by a non-cleavable thioether linker (*N*-succinimidyl-4-(*N*-maleimidomethyl) cyclohexane-1-carboxylate; SMCC attached to anti-ErbB2 antibody trastuzumab and cytotoxic maytansinoid payload DM1 [63]. In Belantamab mafodotin, the anti-BCMA antibody has been conjugated to MMAF via a non-cleavable protease-resistant maleimidocaproyl (mc) linker [64].

### 2.5. Antibody–Drug Conjugation

The conjugation of antibody and the cytotoxic payload can alter the pharmacokinetics and therapeutic index of antibody–drug conjugates. The conventional drug conjugation usually occurs on the mAb backbone either via alkylation or acylation of lysine sidechains as used in gemtuzumab ozogamicin and trastuzumab emtansine or via reduction of disulfide bonds that can liberate cysteine residues to be attached to linkers as utilized in brentuximab vedotin (Figure 1) [65]. These conventional drug conjugation strategies are random and can produce a heterogeneous mixture of ADCs with a different number of drugs to antibody ratio (DAR) leading to variable pharmacokinetics, efficacy and safety profiles [65,66]. The DAR may vary between zero and eight cytotoxic payloads per antibody. Although high DAR can produce more potent ADCs, it can also result in destabilization, aggregation, increased off-target toxicity, and enhanced drug clearance from systemic circulation [67,68]. Hence, there is more interest in site-specific conjugation (SSC) that can produce more homogenous ADCs. These strategies include insertion of engineered cysteine residues, insertion of unnatural amino acids in the antibody sequence or enzymatic conjugation through glycotransferases and transglutaminases [66,69,70,71]. Some ADCs with site-specific conjugation in phase I clinical trials are MEDI2228, PF-06804103 and TR1801-ADC (Table 3) [72].

## 3. Mechanism of Action of ADC

**Figure 2:**
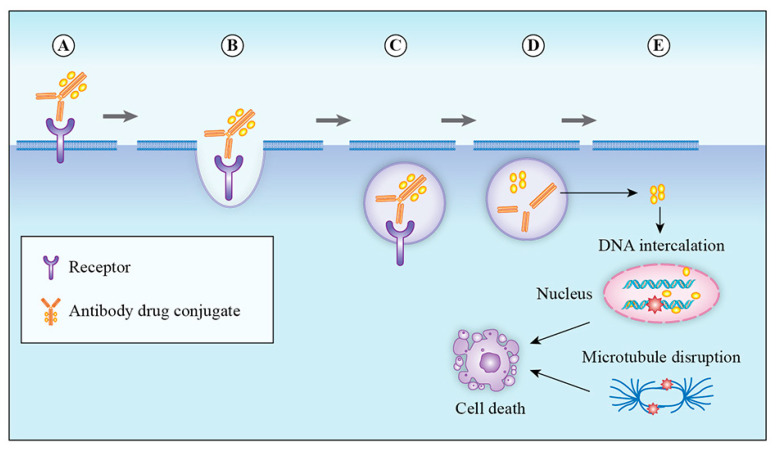
Mechanism of action: (**A**) binding of ADC therapy to target cell surface antigen; (**B**) the antibody–drug conjugate undergoes receptor-mediated endocytosis; (**C**) the antigen–antibody–drug complex is delivered into the lysosomal compartment; (**D**) degradation of the antigen–antibody–drug complex occurs in an acidic and proteolytic enzyme-rich environment, resulting in the intracellular release of the drug; (**E**) intracellular release of the cytotoxic compound, resulting in cell death.

ADCs have poor oral bioavailability and are administered intravenously to prevent degradation by digestive enzymes. After binding to its target, the ADC–antigen complex is typically internalized via clathrin- or caveolae-mediated endocytosis or via pinocytosis [73]. Internalization results in inward budding of the cell membrane to form an early endosome (Figure 2) [74]. Early endosomes mature into late endosomes before fusing with lysosomes. For ADCs with cleavable linkers, the cleavage mechanisms (e.g., hydrolysis, proteolytic cleavage or reductive cleavage) occur in early or late endosomes; however, ADCs with non-cleavable linkers require complex proteolytic cleavage in lysosomes by cathepsin B and plasmin [74]. After cleavage, the cytotoxic payload is released from lysosomes into the cytoplasm. The cytotoxic payloads either target DNA and induce single- or double-stranded DNA breaks or disrupt microtubules, resulting in apoptosis or other means of cell death [75]. It is worth mentioning that ATP-dependent proton pumps in early endosomes establish an acidic environment that creates an interaction between the mAb component of the ADC and the human neonatal Fc receptor (FcRns). ADCs bound to FcRns are recycled back to the cell surface where a physiologic pH of 7.4 releases ADCs from the FcRn. This recycling mechanism prevents normal cell death in the event of misdelivery and prolongs the half-life of antibodies [19].

Some ADCs also act by bystander killing. Bystander killing occurs when neighbouring cells which themselves may not express target antigen are killed by cytotoxic payloads. These cytotoxic payloads are either expelled from cells that express the ADC target antigen following internalization and degradation of the ADC or are released within the tumor microenvironment and do not require ADC internalization. Bystander killing depends on various factors including the extent of ADC internalization, presence of cleavable or non-cleavable linker and properties of cytotoxic payloads [76]. Some ADCs also activate the complement system via antibody dependent cellular toxicity (ADCC), complement-dependent cytotoxicity (CDC) or antibody dependent cellular phagocytosis (ADCP), resulting in infiltration of tumors by immune cells [77,78,79].

## 4. Clinically Approved ADCs

A total of nine ADCs have so far received regulatory approval by the FDA for use in the USA and four ADCs have been approved by the European Medicines Agency (EMA) (Table 2).

### 4.1. Brentuximab Vedotin

Brentuximab vedotin (SGN-35, Adcetris) is an antibody–drug conjugate composed of an anti-CD30 monoclonal antibody linked to tubulin inhibitor MMAE. It not only exerts its anti-cancer activity by binding directly to CD30+ lymphocytes but also has other mechanisms of action including antibody dependent cellular phagocytosis and bystander killing [55].

Brentuximab vedotin (BV) received accelerated FDA approval in 2011 for relapsed or refractory systemic anaplastic large-cell lymphoma (ALCL). ALCL is an aggressive subtype of T cell lymphoma characterized by the uniform expression of CD30. FDA approval was based on a single-arm phase II study when BV induced objective response (OR) in 86% of patients and complete remission (CR) in 57% of patients with relapsed or refractory systemic ALCL after at least one prior therapy [80]. BV is also approved for the treatment of relapsed or refractory Hodgkin’s lymphoma. A meta-analysis of six studies determined an OR of 61% and a CR of 38% as a single agent [81]. BV is also effective when administered in combination with other chemotherapeutic agents. In a single-arm phase I/II study, when administered with bendamustine, BV demonstrated a higher OR of 92.5% and a CR of 73.6% in patients with relapsed or refractory Hodgkin’s lymphoma [82]. Brentuximab vedotin has also shown activity in CD30+ peripheral T cell lymphoma (PTCL). In a phase II study, single-agent BV had an OR of 41% and a CR of 24% in patients with relapsed/refractory CD30+ PTCL [83]. In 2017, BV received FDA approval for treatment of patients with refractory primary cutaneous ALL or CD30+ mycosis fungoides based on a phase III ALCANZA trial that showed an OR of 56.3% that lasted for at least four months vs. 12.5%, resulting in a between-group difference of 43.8% [95% confidence interval (CI) 29.1–58.4; *p* < 0.0001]. In this phase III study, patients were randomized 1:1 to receive BV or treatment of physician’s choice (oral methotrexate, oral bexarotene) for up to 48 weeks [84]. In 2018, brentuximab vedotin was approved for the treatment of patients with previously untreated stage III or IV classical Hodgkin’s lymphoma in combination with chemotherapy and in patients with previously untreated systemic ALCL or CD30+ PTCLs based on ECHELON-1 and ECHELON-2 trials [85,86]. In ECHELON-1 phase III trial, patients with treatment-naïve stage III or stage IV Hodgkin’s lymphoma were assigned to receive either BV, doxorubicin, vinblastine and dacarbazine (A+AVD) or doxorubicin, bleomycin, vinblastine and dacarbazine (ABVD). The 2 year progression-free survival (PFS) rates in A+AVD and ABVD groups were 82.1% versus (vs.) 77.2% [Hazard ratio (HR) 0.77, 95% CI 0.60–0.98; *p* = 0.04). The interim 2 year overall survival rates for A+AVD vs. ABVD were 96.6% vs. 94.2% (HR 0.73, 95% CI, 0.45–1.18; *p* = 0.20) [85]. In ECHELON-2 phase III trial, treatment-naïve CD30+ PTCL patients were randomly assigned to receive either BV, cyclophosphamide, doxorubicin and prednisolone (A+CHP) or cyclophosphamide, doxorubicin, vincristine and prednisolone (CHOP). The median progression-free survival (mPFS) in the BV group was 48.2 months vs. 20.8 months in the CHOP group (HR 0.71, 95% CI 0.54–0.93, *p* = 0.0110). The median overall survival was not reached for both groups. However, improvements in overall survival were noted (HR 0.66, 95% CI 0.46–0.95, *p* = 0.024) [86]. Toxicities with BV include infusion reactions, myelosuppression, gastrointestinal haemorrhage, perforation, acute pancreatitis, progressive multifocal leukoencephalopathy and peripheral sensory neuropathy [80,87,88].

Currently, brentuximab vedotin is being investigated in 83 active phase I–III clinical trials registered on ClinicalTrials.gov in various haematological malignancies in different treatment combinations. Clinical trials worth mentioning are in relapsed or refractory Hodgkin’s lymphoma as an alternative to autologous stem cell transplant (NCT0347133), treatment-naïve Hodgkin’s lymphoma along with chemotherapy vs. immunotherapy (NCT03907488), and diffuse large B cell lymphoma along with lenalidomide and rituximab (NCT04404283).

### 4.2. Trastuzumab Emtansine

Trastuzumab emtansine (T-DM1, Kadcyla) is an ADC, in which anti-ErbB2 (also called anti-HER2) monoclonal antibody trastuzumab is conjugated to microtubule inhibitor DM1 [89].

The pivotal phase III EMELIA trial which led to FDA approval of T-DM1 in 2013 showed an improved mPFS of 9.6 months with T-DM1 vs. 6 months with lapatinib plus capecitabine (HR 0.65, 95% CI 0.55–0.77; *p* < 0.001) and median overall survival (mOS) of 30.9 vs. 25.1 months (HR 0.68, 95% CI 0.55–0.85; *p* < 0.001) in patients with ErbB2-positive advanced breast cancer previously treated with trastuzumab and a taxane [29,54]. T-DM1 was compared with the treatment of physician’s choice in another phase III TH3RESA study in ErbB2-positive advanced breast cancer patients previously treated with two or more ErbB2-directed regimens, including trastuzumab. This trial showed an improved mPFS of 6.2 vs. 3.3 months (HR 0.53, 95% CI 0.42–0.66; *p* < 0.0001) and a mOS of 22.7 vs. 15.8 months (HR 0.68, 95% CI 0.54–0.85; *p* = 0.0007) [90]. The most common adverse events in patients receiving T-DM1 are fatigue, nausea, musculoskeletal pain, thrombocytopenia and constipation [54].

At present, T-DM1 is under investigation in 36 active phase I–III clinical trials registered on ClinicalTrials.gov in ErbB2-positive breast cancer, lung cancer, colorectal cancer and other solid tumors in various combinations. T-DM1 along with either tucatinib, palbociclib or afatinib is being investigated in advanced ErbB2-positive breast cancer patients (NCT03975647, NCT03560696, and NCT04158947). T-DM1 with osimertinib is being evaluated in EGFR mutant lung cancer (NCT03784599) and T-DM1 monotherapy is being tested in advanced ErbB2-positive colorectal cancer patients progressing after trastuzumab and lapatinib (NCT03418558).

### 4.3. Inotuzumab Ozogamicin

Inotuzumab ozogamicin (Besponsa) is an ADC composed of anti-CD22 monoclonal antibody linked to a semi-synthetic derivative of calicheamicin Calich-DMH. Calicheamicin is a DNA-damaging cytotoxic agent derived from soil bacterium *Micromonospora echinospora* [58].

In 2017, Inotuzumab ozogamicin (InO) gained FDA approval for treatment of relapsed or refractory CD22+ acute lymphoblastic leukaemia (ALL), based on phase III clinical trial INO-VATE ALL, that showed significant improvement in mPFS (5.0 vs. 1.8 months, HR 0.45, 95% CI 0.34–0.61; *p* < 0.001) and mOS (7.7 vs. 6.7 months, HR 0.77, 95% CI 0.58–1.03, *p* = 0.04) with InO as compared to investigator’s choice standard intensive chemotherapy [37]. The most commonly observed adverse events with inotuzumab ozogamicin are myelosuppression, increased transaminases, headache, nausea and fatigue [37]. It is also associated with fatal and life-threatening sinusoidal obstruction syndrome also called veno-occlusive disease, which occurred in patients with relapsed or refractory ALL who underwent haematopoietic stem cell transplant (HSCT) after inotuzumab ozogamicin treatment [37,91].

Investigators are eagerly awaiting results of 19 active phase I–III clinical trials registered on ClinicalTrials.gov that are testing inotuzumab ozogamicin in acute lymphoblastic leukaemias, including evaluating InO in the first-line setting along with chemotherapy (NCT03150693), with blinatumomab in B lineage ALL (NCT03739814) and comparing different dosing schedules (NCT03677596).

### 4.4. Gemtuzumab Ozogamicin

Gemtuzumab ozogamicin (Mylotarg) is an anti-CD33 antibody conjugated with calicheamicin derivative (N-acetyl gamma-calicheamicin-dimethyl hydrazide) payload.

In 2000, gemtuzumab ozogamicin (GO) gained FDA accelerated approval for treatment of CD 33+ acute myeloid leukaemia (AML) [92]. However, GO was withdrawn from the market afterwards when post-marketing trials showed that it did not improve survival and was associated with significant toxicities including fatal anaphylaxis, adult respiratory distress syndrome and veno-occlusive disease [92,93]. In 2017, gemtuzumab ozogamicin was reapproved from the FDA based on new data showing efficacy at a lower dose and fractionated dosing schedule. In the ALFA-0701 study, a phase III trial undertaken in patients with untreated AML, 140 patients were assigned to low fractionated-dose GO added to standard first-line chemotherapy (daunorubicin and cytarabine) group, and 140 patients were assigned to receive standard first-line chemotherapy (daunorubicin and cytarabine). Median overall survival was significantly better in patients who received GO as compared to the control group who only received standard treatment (34 vs. 19.2 months, HR 0.69, 95% CI 0.49–0.98; *p* = 0.0368) [94]. Grade 3 and 4 adverse haematological and non-haematological adverse events were increased in patients who were treated with GO, without an increase in the risk of death from toxicity [94]. In another phase III clinical trial, GO significantly improved mOS (4.9 vs. 3.6 months, HR 0.69, 95% CI 0.53–0.90; *p* = 0.005) when compared with best supportive care in older patients with newly diagnosed AML [95]. In this study, the rates of serious adverse events were similar in both groups, and no excess mortality was observed with GO [95].

Currently, there are 31 active phase I–III clinical trials registered on ClinicalTrials.gov investigating gemtuzumab ozogamicin in AML, acute promyelocytic leukaemia and myelodysplastic syndromes in various dosing schedules and along with other chemotherapy combinations.

### 4.5. Polatuzumab Vedotin

Polatuzumab vedotin (Polivy) is an ADC composed of an anti-CD79b monoclonal antibody linked to tubulin inhibitor MMAE.

In a multicenter phase Ib/II clinical trial among patients with relapsed or refractory diffuse large B cell lymphoma (DLBCL), addition of polatuzumab vedotin to anti-CD 20 antibody rituximab and chemotherapy bendamustine showed improved mPFS (9.5 vs. 3.7 months, HR 0.36, 95% CI 0.21–0.63; *p* < 0.001) and mOS (12.4 vs. 4.7 months, HR 0.42, 95% CI 0.24–0.75; *p* = 0.002) [48]. Polatuzumab vedotin received accelerated approval from the FDA in 2019 for treatment of relapsed or refractory DLBCL. The most common side effects noted with polatuzumab vedotin are myelosuppression, peripheral neuropathy. Polatuzumab vedotin has also been associated with progressive multifocal leukoencephalopathy (PML) [48].

At present, polatuzumab vedotin is under investigation in eight active phase I–III clinical trials registered on ClinicalTrials.gov in haematological malignancies. These studies are further investigating polatuzumab vedotin for the treatment of Hodgkin’s lymphoma (NCT04491370), first-line treatment for DLBCL (NCT02611323, NCT04231877), first-line treatment for double- or triple-hit lymphoma (NCT04479267), and chronic lymphocytic leukaemia (NCT01290549).

### 4.6. Trastuzumab Deruxtecan

Trastuzumab deruxtecan (DS-8201a, Enhertu) is an ADC, composed of an anti-ErbB2 monoclonal antibody, with the same amino acid sequence as trastuzumab, conjugated DXd (DX-8951 derivative). Trastuzumab deruxtecan has a higher drug to antibody ratio as compared to T-DM1 (8 vs. 3 to 4) [45].

In a multicenter single-arm phase II study of patients with previously treated ErbB2-positive metastatic breast cancer, trastuzumab deruxtecan showed OR in 60.9% of patients with a mPFS of 16.4 months (95% CI, 12.7–not reached) [45]. Trastuzumab deruxtecan was approved by the FDA in 2019, for treatment of ErbB2+ advanced breast cancer patients who have received prior ErbB2-directed therapies in the metastatic setting. The most common toxicities noted were myelosuppression and nausea. Trastuzumab deruxtecan was also associated with interstitial lung disease [45].

Currently, trastuzumab deruxtecan is under investigation in 15 active phase I–III clinical trials registered on ClinicalTrials.gov in various solid organ malignancies including ErbB2-expressing non-small-cell lung cancer (NCT03505710), ErbB2 low breast cancer (NCT03734029), ErbB2-positive gastric cancer (NCT04014075) and with immunotherapy combination nivolumab in advanced breast and urothelial cancers (NCT03523572).

### 4.7. Enfortumab Vedotin

Enfortumab vedotin (ASG-22ME, Padcev) is an ADC composed of nectin-4-directed monoclonal antibody with tubulin inhibitor cytotoxic payload MMAE [49].

Enfortumab vedotin (EV) received accelerated approval from the FDA in December 2019 for treatment of patients with relapsed or refractory locally advanced or metastatic urothelial cancer. It was the first ADC directed against nectin-4 to receive FDA approval. FDA approval was based on a pivotal single-arm phase II clinical trial in patients with advanced or metastatic urothelial cancer previously treated with platinum-based chemotherapy and an immune checkpoint inhibitor. The trial showed an OR of 44% and a CR of 12%, with a mPFS of 5.8 months and a mOS of 11.7 months [96]. The main toxicities associated with enfortumab vedotin are fatigue, alopecia, decreased appetite and peripheral sensory neuropathy. Febrile neutropenia was the most common serious adverse event [96].

Currently, enfortumab vedotin is under investigation in seven active phase I–III clinical trials registered on ClinicalTrials.gov. Enfortumab vedotin is compared to chemotherapy in a phase 1 clinical trial (NCT03474107) in patients with advanced urothelial cancer. The combination of enfortumab vedotin and immunotherapy pembrolizumab is also being evaluated in another phase III clinical trial (NCT04223856) in patients with advanced urothelial cancer. EV is also being tested in other solid organ tumors that express nectin-4 (NCT02091999).

### 4.8. Sacituzumab Govitecan

Sacituzumab govitecan (IMMU-132, Trodelvy) is an ADC that targets tumor-associated calcium signal transducer 2 (TROP-2) for the selective delivery of SN-38 [43].

It has been recently approved for patients with metastatic triple-negative breast cancer (mTNBC), who have received at least two systemic therapies for metastatic disease. In a single-arm phase I/II trial of mTNBC patients, sacituzumab govitecan resulted in an OR of 33.3% (3 complete and 33 partial responses), with a mPFS of 5.5 months and a mOS of 13 months [97]. The most common adverse events noted with this ADC are myelosuppression and diarrhoea [97].

Currently, sacituzumab govitecan is under investigation in seven active phase I–III clinical trials registered on ClinicalTrials.gov. Sacituzumab govitecan is being evaluated in endometrial cancer (NCT04251416), in patients with glioblastoma (NCT03995706) and in patients with urothelial cancer (NCT03547973). Sacituzumab govitecan and pembrolizumab combination is under investigation in hormone receptor-positive and ErbB2-negative advanced breast cancer (NCT04448886), and in advanced TNBC (NCT04468061). It’s combination with talazoparib (NCT04039230) is being tested in metastatic breast cancer patients.

### 4.9. Belantamab Mafodotin

Belantamab mafodotin (GSK2857916, Blenrep) is an ADC that targets BCMA, a cell surface B cell maturation antigen expressed on multiple myeloma cells [98]. BCMA promotes growth and survival of plasma cells, and its serum levels correlate with response to therapy and overall survival in patients with multiple myeloma [99].

Belantamab mafodotin has received recent FDA approval as a monotherapy treatment for patients with relapsed or refractory multiple myeloma who have received at least four prior therapies including an anti-CD38 monoclonal antibody, a proteasome inhibitor and an immunomodulatory agent. FDA approval was based on the phase II DREAMM-2 study. In this two-arm study, heavily pretreated patients with multiple myeloma received 2.5 or 3.4 mg/kg of belantamab mafodotin every three weeks. The overall response rate was 31% (97.5% CI 20.8–42.6) in patients in the 2.5 mg/kg cohort and 34% (97.5% CI 23.9–46.0) in patients in the 3.4 mg/kg cohort. The most common adverse events reported were keratopathy, decreased visual acuity blurred vision, infusion-related reactions, anaemia and thrombocytopenia [100].

Belantamab mafodotin is currently being evaluated in five active phase I–III clinical trials registered on ClinicalTrials.gov in multiple myeloma. It is under investigation as monotherapy (NCT04126200), with chemotherapy (NCT04162210), with or without VRd bortezomib, lenalidomide and dexamethasone (NCT04091126) and with proteasome inhibitor bortezomib (NCT04246047).

## 5. Promising ADCs in Clinical Development

Despite the approval of only nine ADCs, there has been significant improvement in ADC design. At present, there are more than 80 ADCs under clinical development worldwide (Table 3) [5,6]. ADCs are at a frontier for the next generation of systemic therapeutic options for cancer patients. Lessons learned from clinical development of approved ADCs are informing scientists and clinicians for the selection of appropriate target antigens, monoclonal antibodies, liners and cytotoxic payloads.

**Table 3:** Antibody–drug conjugates in clinical development.

Payload	Target Antigen	Antibody–Drug Conjugates	Antibody	Linker	Lead Indication	Trial Phase	ClinicalTrials.gov Identifier	Reference
AGD-0182	FLT3	AGS62P1 (ASP1235)	Human IgG1	Non-cleavable linker	Acute myeloid leukaemia (AML)	I	NCT02864290	[101]
Amberstatin-269	FLT3	AGS-62P1	Humanized IgG1	Cleavable linker	AML	I	NCT02864290	
Auristatin-0101	ErbB2	PF-06804103	Humanized IgG1	Protease-cleavable linker	Advanced solid tumors	I	NCT03284723	[102]
	PTK7	Cofetuzumab pelidotin PF-7020	Humanized IgG1	Cleavable maleimidoca- proyl -valine-citrulline (mc-vc) PABC linker	Non-small-cell lung cancer (NSCLC), advanced solid tumors	I	NCT04189614 NCT02222922	[103]
Auristatin derivative	IGF-1R	W0101	Humanized IgG1	Non-cleavable maleimidoca- proyl (mc) linker	Advanced solid tumors	I/II	NCT0331638	[104]
Auristatin	ErbB2	ZW49	Biparatopic IgG	Protease-cleavable linker	ErbB2-expressing cancers	I	NCT03821233	[105]
Auristatin F	5T4	ASN-004	Humanized scFvFc antibody	Non-cleavable linker	Advanced solid tumors	I	NCT04410224	
Auristatin F-HPA (DolaLock)	SLC34A2 (NaPi2b)	XMT-1592	Humanized IgG1	Protease-cleavable linker	NSCLC, ovarian cancer	I/II	NCT04396340	
	SLC34A2 (NaPi2b)	XMT-1536	Humanized IgG1	Protease-cleavable linker	NSCLC, ovarian cancer	I	NCT03319628	[106]
Batansine	ErbB2	BAT8001	Humanized IgG1	Non-cleavable linker	Metastatic breast cancer	I	NCT04189211 NCT04151329	[107]
	TROP2	BAT8003	IgG1	Non-cleavable linker	Advanced epithelial cancer	I	NCT03884517	[108]
Dolastatin analogue	AG7	ABGn-107	Humanized IgG	Cleavable linker	Gastric, colorectal, pancreatic, or biliary cancer	I	NCT02908451	[109]
DM1	CD30	F0002-ADC	Chimeric IgG1κ	Non-cleavable SMCC linker	Hematologic malignancies	I	NCT03894150	[110]
	CD37	Naratuximab emtansine (IMGN-529, DEBIO 1562)	Humanized IgG1	Non-cleavable SMCC linker	Non-Hodgkin’s lymphoma (NHL)	I	NCT01534715	[111]
	CD56	Lorvotuzumab Mertansine (LM, IMGN-901)	Humanized IgG1	Disulfide linker	Multiple myeloma, small-cell lung cancer (SCLC), Merkel cell, ovarian cancer	I/II	NCT02452554	[112,113,114]
	EGFR	AVID100	IgG	Non-cleavable SMCC linker	Advanced epithelial carcinomas	I/II	NCT03094169	[115]
	ErbB2	B003	Humanized IgG1	Thioether linker	Metastatic breast cancer	I	NCT03953833	
DM4	CD205	MEN1309 (OBT076)	Humanized IgG1	Cleavable SPDB linker	Solid tumors, breast cancer, NHL	I	NCT04064359	[116]
	CD138	Indatuximab ravtansine (BT062)	Chimeric IgG4	Cleavable disulfide linker	Multiple myeloma	I/IIa	NCT01638936	[117,118]
	CD166	CX-2009	Probody	Cleavable SPDB linker	Solid tumors	I/II	NCT03149549	[119]
	CEACAM5	SAR408701	Humanized IgG1	Cleavable linker	Solid tumors	III	NCT04154956	[120]
	Folate receptor α (FRα)	Mirvetuximab soravtansine (IMGN-853)	Humanized IgG1	Cleavable SPDB linker	NSCLC, ovarian cancer	I/II/ III	NCT01609556 NCT02631876 NCT03552471 NCT02496890 NCT03832361	[121]
	Mesothelin (MSLN)	Anetumab ravtansine (BAY 94-9343)	Human IgG1	Cleavable SPDB linker	Mesothelioma, Solid tumors	II	NCT03832361 NCT04296890 NCT03552471 NCT03023722 NCT04209855 NCT02996825	[122,123]
DUBA (Seco-duocarmycin-hydroxy-benzamide-azainodole)	ErbB2	Trastuzumab duocarmazine (SYD985)	Humanized IgG1	Cleavable linker	Endometrial carcinoma, metastatic breast cancer,	II/III	NCT04205630 NCT03262935	[39]
	B7-H3	MGC018	Humanized IgG1	Cleavable valine-citrulline linker	Advanced solid tumors	I/II	NCT03729596	[124]
Duocarmycin analogue	5T4	SYD1875	Humanized IgG1	Cleavable valine-citrulline-seco linker	Advanced solid tumors	I/II	NCT04202705	
Dxd (DX-8951)	B7-H3	DS-7300a	Humanized IgG1	Cleavable linker	Advanced solid tumors	I	NCT04145622	
	ErbB3	U3-1402	Humanized IgG1	Cleavable linker	NSCLC, breast cancer, colorectal cancer	I/II	NCT03260491, NCT02980341, NCT04479436	[125]
	GPR20	DS-6157a	Unknown	Cleavable Gly-Gly-Phe-Gly (GGFG) linker	Advanced gastrointestinal stromal tumor	I	NCT04276415	[126]
	TROP-2	DS-1062a	Humanized IgG1	Cleavable linker	NSCLC, Advanced solid tumors	I	NCT04526691 NCT03401385	[127]
Eribulin	FRα	MORAb-202	Humanized IgG1	Cleavable mal-PEG2-vc-PABC	Solid tumors	I	NCT03386942	[128]
IGN (Indolino-benzodiazepine dimer)	CD123	IMGN 632 Orphan drug designation	Humanized IgG1	Cleavable peptide linker	AML, Acute lymphoblastic leukaemia (ALL)	I/II	NCT03386513	[129]
MMAE	AXL	Enapotamab vedotin (HuMax-AXL-ADC)	Human IgG1	Cleavable mc-vc linker	Solid tumors	I/II	NCT02988817	[130]
	AXL	BA3011 (CAB-AXL-ADC)	Humanized IgG1	Cleavable linker	Advanced solid tumors	I/II	NCT03425279	[131]
	CD71	CX-2029	Probody	Protease-cleavable mc-vc-PAB linker	DLBCL, Solid tumors	I/II	NCT03543813	[132]
	CD228	SGN-CD228A	Humanized IgG1	Protease-cleavable linker	Advanced solid tumors	I	NCT04042480	[133]
	C-Met	ABBV-399 Telisotuzumab vedotin	Humanized IgG1	Cleavable mc-vc linker	Non-small-cell lung cancer	II	NCT03539536	[134]
	ErbB2	Disitimab vedotin (RC48-ADC)	Humanized IgG1	Protease-cleavable linker	Metastatic breast cancer with low ErbB2 expression	III	NCT04400695	[135,136]
	ErbB2	ALT-P7	Humanized IgG1	Unknown	Breast cancer	I	NCT03281824	[137]
	ErbB2	MRG002	IgG	Unknown	Breast cancer, gastric cancer	I/II	NCT04492488	[138]
	EGFR	MRG003	IgG	Unknown	Solid tumors	I	CTR20180310	
	Globo H	OBI-999 Orphan drug approval from the FDA	Humanized IgG1	Cleavable thiobridge linker	Advanced solid tumors	I/II	NCT04084366	[139]
	Integrin beta-6	SGN-B6A	IgG	Unknown	Advanced solid tumors	I	NCT04389632	
	ROR1	VLS-101 (UC-961ADC3)	Humanized IgG1	Unknown	Advanced breast cancer, NSCLC, haematological malignancies	I	NCT04504916	[140]
	SLC39A6 (LIV-1)	Ladiratuzumab vedotin (SGN-LIV1A)	Humanized IgG1	Cleavable mc-vc linker	NSCLC, head and neck cancer, gastric cancer, breast cancer	I/II	NCT04032704 NCT03310957 NCT01969643	[141]
	Tissue factor (TF, CD142)	Tisotumab vedotin (HuMax-TF-ADC)	Human IgG1	Protease-cleavable mc-vc-PAB linker	Advanced ovarian cancer	II	NCT03657043	[142]
MMAF	ErbB2	ARX788	IgG1	pAcF linker	ErbB2-expressing cancers	I	NCT03255070	[143]
	ErbB2	FS-1502 (LCB14-0110)	Humanized IgG1	Unknown	ErbB2-positive advanced solid tumors	I	NCT03944499	
MMAF derivative (Duostatin-5)	ErbB2	A166	Humanized IgG1	Unknown	ErbB2-expressing tumors	I/II	NCT03602079	[144]
Maytansinoid	CD22	TRPH-222	Humanized IgG	Non-cleavable linker	B cell lymphoma	I	NCT03682796	[145]
PBD	BCMA	MEDI2228	Humanized IgG1	Cleavable protease linker	Multiple myeloma	I	NCT03489525	
	c-MET	TR 1801-ADC (MT-8633)	Humanized IgG2	Cleavable Val-Ala linker	c-MET-expressing tumors	I	NCT03859752	
	CD19	Loncastuximab tesirine (ADCT-402)	Humanized IgG1	Cleavable Valine-Ala linker	NHL	III	NCT04384484	[146]
	CD25	Camidanlumab tesirine (ADCT-301)	Humanized IgG1	Cleavable linker	AML, ALL, HL, NHL, solid tumors	I/II	NCT04052997 NCT03621982	[147]
	EGFR	ABBV-321 Serclutamab talirine	IgG		Solid tumors	I	NCT03234712	[148]
	GCC	TAK-164	Human IgG	Cleavable peptide linker	Gastrointestinal malignancies	I	NCT03449030	[149]
SC236	CD74	STRO-001	Human IgG1	Non-cleavable DBCO linker	NHL, B cell malignancies	I	NCT03424603	[150]
SC209	FR α	STRO-002	Human IgG1	Cleavable linker	Ovarian and endometrial cancers	I	NCT03748186	[151]
SG3249 (PBD derivative)	CD22	ADCT-602 (Epratuzumab tesirine)	Humanized IgG1	Cleavable Val-Ala linker	B cell acute lymphoblastic leukaemia	I/II	NCT03698552	
SHR152852	c-MET	SHR-A1403 (HTI-1066)	Humanized IgG2	Non-cleavable linker	Advanced solid tumors	I	NCT03856541	[152]
PNU-159682	ROR1	NBE-002	IgG	Unknown	Advanced solid tumors, triple-negative breast cancer	I	NCT04441099	
Belotecan-derived payload	TROP2	SKB-264	IgG	Unknown	Advanced solid tumors	I/II	NCT04152499	
TLR7/TLR8 agonist	ErbB2	BDC-1001	Humanized IgG1	Unknown	ErbB2-positive advanced solid tumors	I	NCT04278144	
TLR8 agonist	ErbB2	SBT6050	IgG	Unknown	ErbB2-positive advanced solid tumors	I	NCT04460456	
Unknown	ROR2	BA 3021 (CAB-ROR2-ADC)	Humanized IgG1	Unknown	Advanced solid tumors	I/II	NCT03504488	
Unknown	CD46	FOR-46	Humanized IgG1	Unknown	Multiple myeloma, prostate cancer	I	NCT03650491 NCT03575819	
Unknown	Unknown	ABBV-011	IgG	Unknown	Small-cell lung cancer	I	NCT0369194	
Unknown	Unknown	ABBV-155	IgG	Unknown	Solid tumors	I	NCT03595059	
Unknown	ErbB2	DP303c	IgG	Unknown	ErbB2-positive advanced solid tumors	I	NCT04146610	
Unknown	BCMA	CC-99712 (BCMA-ADC)	IgG	Unknown	Multiple myeloma	I	NCT04036461	
Unknown	ErbB2	GQ1001	IgG	Unknown	ErbB2-positive advanced solid tumors	I	NCT04450732	
Unknown	ErbB2	BB-1701	IgG	Unknown	ErbB2-positive advanced solid tumors	I	NCT04257110	
Unknown	ErbB2	SHR-A1811	IgG	Unknown	ErbB2-positive advanced solid tumors	I	NCT04513223 NCT04446260	
Unknown	CCR7	JBH492	IgG	Unknown	CLL, NHL	I	NCT04240704	

## 6. Mechanisms of Resistance to Antibody–Drug Conjugate Therapies

**Figure 3:**
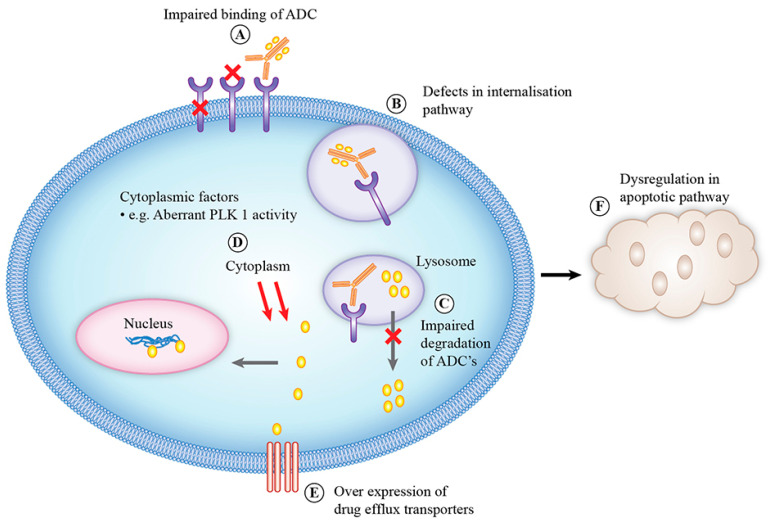
Mechanism of resistance: (**A**) impaired binding of the ADC to the target antigen by antigen downregulation, loss of antigen expression or mutations in the antigen; (**B**) defects in the internalization pathway and reduced cell surface trafficking; (**C**) impaired degradation of ADCs in lysosomes due to reduced lysosomal proteolytic or acidification function or loss of lysosomal transporter expression inhibiting the release of cytotoxic payload lysosomes to the cytoplasm; (**D**) cytoplasmic factors, e.g., aberrant polo-like kinase 1 activity preventing mitotic arrest or due to defective cyclin B1 induction; (**E**) the overexpression of drug efflux transporters; (**F**) dysregulation in the apoptotic pathway through the deficient activity of proapoptotic proteins Bak and Bax or the overexpression of antiapoptotic proteins BCl-2 and Bcl-x.

As with all anti-cancer therapeutics, resistance is a significant problem with the use of ADCs. The mechanisms of resistance to ADCs are complex and are influenced by various factors, either arising from each component of the ADC or from a pathway of the mechanism of action of ADCs (Figure 3) [153]. One major class of resistance mechanisms affects drug delivery. Binding of the ADC to the target antigen may be hampered by downregulation of antigen, loss of antigen expression or mutations in the antigen affecting its recognition by monoclonal antibody. Reduced ErbB2 expression has been identified as an acquired mechanism of resistance to T-DM1 in T-DM1 resistance cell lines [154]. Loss of CD30 antigen expression has been reported as the cause of resistance to brentuximab vedotin in a patient with anaplastic large-cell lymphoma [155]. Presence of ligands for antigens can also cause resistance to ADC. Neuregulin is a ligand for ErbB3, and its presence leads to ErbB2 and ErbB3 heterodimerization, resulting in reduced cytotoxicity of T-DMI in breast cancer cell lines [156]. After binding to the target antigen, the ADC–antigen complex is internalized via clathrin- or caveolae-mediated endocytosis or via pinocytosis [73]. This internalization process can be altered by defects in the internalization pathway and reduced cell surface trafficking. T-DM1-resistant N87 cells internalize ADCs into intracellular caveolin and alter their trafficking to the lysosomes. T-DM1 colocalization into intracellular caveolin-positive puncta correlated with reduced response to T-DM1 in a panel of ErbB2-positive cell lines [157]. Once inside cells, degradation of ADCs in lysosomes may be impaired by reduced lysosomal proteolytic or acidification function, resulting in failure of cleavage of cytotoxic payload from ADCs. Cell lines resistant to T-DM1 have shown increased lysosomal pH, leading to decreased proteolytic activity and accumulation of T-DM1 in lysosomes [158]. Loss of lysosomal transporter expression, e.g., SLC46A3, can inhibit the release of cytotoxic payload from lysosomes to the cytoplasm. Loss of SLC46A3 expression has been reported as a mechanism of innate and acquired resistance to PBD and DM1 bearing ADCs [159].

Inside the cytoplasm, the expression of the mitotic polo-like kinase 1 (PLK1) has been reported to confer T-DM1 resistance by completing mitosis despite the presence of an abnormal mitotic spindle and thus avoiding mitotic catastrophe by preventing cyclin-dependent kinase 1 (CDK1) activation. Reduced induction of CDK1 results in an inability to halt cell cycle progression [160]. T-DM1 treatment can increase cyclin B1 expression, leading to cell cycle arrest in the G2/M phase, whereas knockdown of cyclin B1 can induce T-DM1 resistance [160,161]. Drug efflux transporters can efflux ADCs from cells, resulting in drug resistance, which is a common mechanism for chemotherapy drug resistance. Drug efflux pumps have been reported for resistance to gemtuzumab ozogamicin, brentuximab vedotin and T-DM1 [153].

Resistance can also result from acquired or innate insensitivity to the payload. Dysregulation in the apoptotic pathways may also contribute to cancer cell sensitivity to ADCs. For example, the deficient activity of proapoptotic proteins Bak and Bax and the overexpression of antiapoptotic proteins BCl-2 and Bcl-x can confer resistance to gemtuzumab ozogamicin [162,163]. Another mechanism of resistance to ADCs is an effect of ADCs on the cell cycle. Silencing of cyclin B, a cell cycle protein that participates in the G2–M transition, can result in resistance to T-DM1 [153]. The heterogeneity of target antigens expressed in tumors may also lead to ADC inability to kill low antigen-expressing cells. The issue of tumor antigen heterogeneity and its evolution over time is compounded by the use of archival tissue for screening for ADC treatment eligibility. Assessment of target antigen expression is pivotal in the preclinical and clinical development of ADCs. Molecular imaging of target antigen expression and tracking of drug delivery in vivo are emerging areas of interest for better patient selection and ADC development.

Researchers are working on various strategies to overcome ADC drug resistance. In order to prevent efflux from cells, the cytotoxic payload of ADCs can be changed to toxins that are poor efflux substrates. Trastuzumab deruxtecan containing a DXd cytotoxic payload can overcome T-DM1 resistance in ErbB2-positive metastatic breast cancer patients [45]. Using a cytotoxic payload with an alternate mechanism of action can also overcome drug resistance. For example, an anti-CD22-NMS249 ADC containing anthracycline as a payload overcame acquired drug resistance in NHL models, that became resistant to anti-CD22 ADC containing MMAE as a cytotoxic payload [164]. As drug efflux transporters efflux hydrophobic compounds more efficiently than hydrophilic compounds, ADCs can be designed using maleimidyl-based hydrophilic linkers. The maleimidyl-based hydrophilic linker PEG_4_Mal has shown promising activity in preclinical studies in drug-resistant models [165]. Switching from a non-cleavable linker to a protease-cleavable mc-vc-PAB linker has overcome T-DM1 resistance in preclinical models [164]. New designs such as bispecific and biparatopic antibodies can increase cellular internalization. MM-302 is a biparatopic ADC which uses the scFv fragment of the monoclonal antibody. Further development of this ADC has now been discontinued owing to disappointing results in a phase II clinical trial for advanced ErbB2-positive breast cancer patients. Various clinical trials are testing a combination of ADCs with immunotherapy to overcome drug resistance and increase clinical response. For example, brentuximab vedotin and nivolumab with or without ipilimumab is in a phase I/II clinical trial NCT01896999 and T-DM1 with pembrolizumab is in a phase I clinical trial NCT03032107.

## 7. Role of Molecular Imaging in the Clinical Development of ADCs

The discovery and development of new drugs is challenging, expensive and time consuming. On average, it takes at least 5–10 years to bring a new drug to market and this costs up to US$1 billion [166,167]. Owing to the dimension of investment involved, and the need for a more efficient process of drug development, there is renewed interest in utilizing molecular imaging for preclinical and clinical development of drugs, including ADCs. Molecular imaging allows the development of imaging probes that can identify target expression or can image drug distribution and confirm in vivo target delivery and normal tissue distribution and pharmacokinetics in real time [168]. Molecular imaging has been successfully used in ADC preclinical studies and in clinical trials in order to determine tumor target expression, drug stability and delivery to the tumor, and interlesional target heterogeneity. These factors are vital in delivering a safe dose while maximizing antitumor effects as well as providing insight into the in vivo properties of these drugs [169,170]. Both SPECT and PET imaging of ADCs have been reported in preclinical and human studies, with the superior resolution and quantitative capabilities of immunoPET showing particular promise [171]. The majority of immunoPET studies with intact ADCs have utilized Zirconium-89 (^89^Zr) due to its suitability for radiochemistry and long half-life (T1/2 = 78.4 h) which facilitates optimal imaging of the biodistribution of intact mAbs.

### 7.1. Preclinical Studies

#### 7.1.1. CD30

Kang et al. [172] radiolabeled brentuximab vedotin with ^89^Zr to enable tracking of BV and determine CD30 expression in three lung cancer models with different levels of CD30 expression. In vitro characterization revealed strong binding affinity between ^89^Zr-Df-BV and CD30 in a cell line with high CD30 expression (Kd 2.07 ± 1.86 nM). PET and biodistribution data also showed that CD30 mediated uptake with a peak value of 9.93 ± 2.70% ID/g in the CD30 high-expressing model 24 h post-injection, while CD30 mice had 5.00 ± 1.56% ID/g at 24 h post-injection, demonstrating different signals in tumors with different levels of CD30 expression.

A novel CD30-targeting ADC comprising of an anti-CD30 antibody conjugated to two molecules of lidamycin (LDM) via a non-cleavable linker has been developed [173]. Anti-CD30-LDM alone or combination with crizotinib has shown significant anti-tumor activity in a CD30+/ALK+ anaplastic large-cell lymphoma model [174]. Anti-CD30-LDM was radiolabeled with ^123^I and demonstrated strong binding capabilities in CD30-positive cells, and a biodistribution study showed a 2-fold higher tumor uptake in CD30+ versus CD30- tumors (4.98 ± 0.99%ID/g vs. 2.75 ± 0.47%ID/g, respectively) [175]. These findings support the potential use of ^123^I-anti-CD30-LDM as a companion diagnostic in the further clinical development of anti-CD30-LDM.

#### 7.1.2. Tomoregulin (TENB2) and Six-Transmembrane Epithelial Antigen of the Prostate-1 (STEAP1)

The transmembrane proteins TENB2 and STEAP1 are commonly overexpressed in prostate cancer [176,177]. The therapeutic efficacy of ADCs targeting these antigens in a panel of patient-derived prostate cancer models was evaluated. In addition, parallel biodistribution and immunoPET studies were performed using corresponding ^111^In and ^89^Zr-radiolabeled mAbs to quantify tumor uptake and establish the relationship between imaging with therapeutic response [178]. In this study, both TENB2 and STEAP1 expression correlated with antibody uptake and ADC efficacy, indicating that this imaging approach may provide crucial information on the suitability of tumor type for ADC treatment.

#### 7.1.3. Mesothelin (MSLN)

Mesothelin is a tumor antigen that is highly expressed in several tumor types and plays a vital role in promoting proliferation and invasion [179]. The efficacy of the mesothelin-targeting ADC, DMOT4039A, an anti-mesothelin antibody (AMA, MMOT0530A) conjugated to MMAE, was evaluated in a range of tumor models with different levels of MSLN expression [170]. Correlative molecular imaging to measure tumor antibody uptake by ^89^Zr immunoPET imaging was also undertaken. ADC efficacy correlated with antigen expression and ^89^Zr-MMOT0530A uptake six days post-injection was highest in tumors with high mesothelin expression. The less responsive xenografts did not show ^89^Zr-AMA uptake despite confirmed mesothelin expression and this was attributed to lower MSLN expression levels and/or potential differences in target internalization. Encouraged by these preclinical findings, ^89^Zr-AMA immunoPET has been translated to the clinic [180].

Another anti-MSLN antibody, MMOT05530A, was radiolabeled with ^89^Zr for PET imaging of MSLN-expressing human pancreatic tumor xenografts. Biodistribution studies showed specific tumor uptake of ^89^Zr-MMOT0530A at 10, 25, and 100 mg. PET data showed similar tumor uptake of ^89^Zr-MMOT0530A that increased over six days, whereas activity in other tissues decreased. Ex vivo biodistribution analyses were consistent with imaging data for both xenograft models

#### 7.1.4. Leucine-Rich Repeat-Containing G Protein-Coupled Receptor 5 (LGR5)

LGR5 is highly expressed in colorectal tumors and marks colon cancer stem cells that drive tumor growth and metastasis [181]. LGR5 overexpression was shown to correlate with poor clinical outcomes [182]. Two different anti-LGR5 monoclonal antibodies, 8F2 and 9G5, were evaluated using ^89^Zr immunoPET to select the optimal mAb for ADC development and tumor imaging [181]. Two LGR5-targeting ADCs were generated by conjugating the anti-LGR5 mAb (anti-LGR5 mAb) to MMAE via a cleavable or non-cleavable chemical linker [183]. Both mAbs demonstrated high-affinity specific binding and rapidly internalized into lysosomes and promoted ADC-induced cytotoxicity in LGR5-overexpressing colon cell lines. ^89^Zr-labeled mAbs retained high affinity and LGR5-dependent uptake in vitro. Biodistribution studies showed specific tumor uptake of ^89^Zr-MMOT0530A at 10, 25, and 100 µg doses. PET data showed that tumor uptake of ^89^Zr-MMOT0530A increased over six days while activity in other tissues decreased. Ex vivo biodistribution analyses conducted on tumoral cell lines were consistent with imaging data from the animal models. Based on the slightly higher potency, the investigators identified 8F2 as the optimal candidate for ADC development and this is also supported by the previous studies [183]. These studies provide a strong rationale for using ^89^Zr immunoPET for the development of LGR5-targeted ADCs, target quantification and selecting patients likely to respond best to LGR5-targeted ADC therapy.

### 7.2. Clinical Studies

The role of molecular imaging in ADC clinical development has been demonstrated using SPECT and PET approaches. These clinical trials have utilized molecular imaging to evaluate the biodistribution and pharmacokinetics of ADCs and to measure target expression and deliverable ADC concentration to a tumor. This information is vital to understanding the in vivo behaviour of ADCs in patients in order to ensure the patient selection and ADC dose is optimized as well as to allow valid assessment of the therapeutic effects of ADCs.

**Figure 4:**
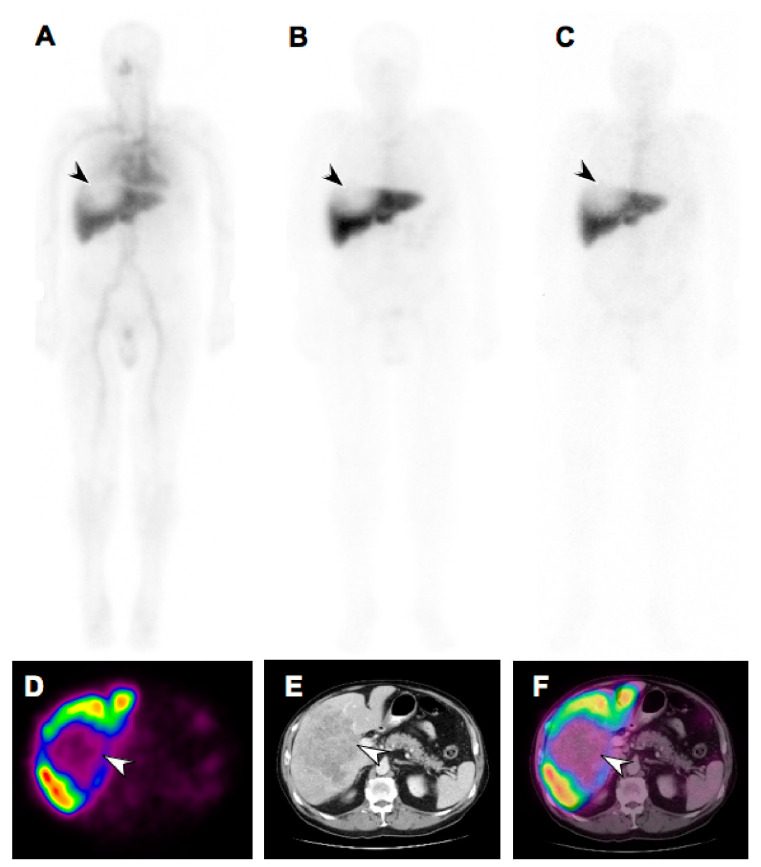
Representative biodistribution pattern of ^111^In-CMD-193. Anterior whole-body γ camera images in patient 106 (1.0 mg/m2 dose cohort) following infusion are shown for day 1 (**A**), day 3 (**B**), and day 8 (**C**). Following infusion of ^111^In-CMD-193, there was initial blood pooling, followed by markedly increased hepatic uptake by day 2 that persisted to day 8. No tumor uptake was apparent in the whole-body γ camera images (arrow) or SPECT (**D**). (**E**), corresponding CT scan shows the large hepatic metastasis, also evident in (**F**), coregistered SPECT/CT scan. Reprinted from the phase I biodistribution and pharmacokinetic study of Lewis Y-targeting immunoconjugate CMD-193 in patients with advanced epithelial cancers [186].

CMD-193 is an ADC which is composed of G193, a humanized anti-Lewis Y (Le^y^) monoclonal antibody based on Hu3S193, conjugated to cytotoxic calicheamicin via an acid-labile AcBut linker [184]. The Le^y^ antigen (CD174) is a cell surface antigen expressed in various malignancies including breast, ovary, pancreas, colon, prostate, multiple myeloma and lung cancer. In preclinical studies, CMD-193 showed regression of Le^y^-expressing human carcinoma xenografts [185]. However, in a phase I dose escalation study of CMD-193 in patients with Le^y^-expressing advanced solid tumors, none of the nine patients showed a radiological response. This was the first clinical study which utilized a radiolabeled ADC (^111^In-CMD-193) in order to evaluate the biodistribution and pharmacokinetics of an ADC [186]. Patients received a single infusion of ^111^In-CMD-193, this was followed by unlabeled CMD-193 infusions every three weeks for the duration of the study. Biodistribution analysis was performed by whole-body gamma camera scans and SPECT over one week following ^111^In-CMD-193 infusion. Biodistribution images revealed that there was a rapid clearance of ^111^In-CMD-193 from blood followed by a marked increase in hepatic uptake, and no significant uptake in tumor (Figure 4). This was in stark contrast to parental hu3S193 monoclonal antibody, which had a long half-life in blood, lower hepatic uptake and higher tumor uptake [187]. This physiochemical change in CMD-193 may have been induced by conjugation of mAb with cytotoxic payload calicheamicin [186]. Based on this study, the clinical development of CMD-193 was discontinued. This clinical trial highlighted the significance of molecular imaging to study the biodistribution and pharmacodynamics of drugs in the early phase of development.

Molecular imaging techniques are also under investigation to measure target expression in order to predict response to ADCs. The ZEPHIR trial was the first study to use the molecular imaging probe ^89^Zr-trastuzumab to measure ErbB2 expression and predict response to T-DM1 [188]. In the ZEPHIR study, ErbB2-positive advanced metastatic breast cancer patients underwent ErbB2-PET (^89^Zr-trastuzumab PET/CT) and FDG-PET/CT followed by FDG-PET/CT after once cycle of T-DM1 and standard CT scans after three cycles of therapy in order to assess response. The primary end point of the study was negative predictive value (NPV) for therapeutic response. Positive predictive value (PPV) and time to treatment failure (TTF) were also measured. The NPV and PPV for ErbB2-PET/CT were 88% and 72% respectively. The NPV and PPV for FDG-PET/CT were 83% and 96%, respectively. Combining ErbB2–PET/CT and FDG–PET/CT accurately predicted morphological response (NPV and PPV of 100%) and distinguished patients with a median TTF of only 2.8 months [n = 12, 95% confidence interval (CI) 1.4–7.6] from those with a TTF of 15 months (n = 25, 95% CI 9.7–not calculable) [188]. This study displayed the potential of molecular imaging as a companion diagnostic tool for ADC therapy in selecting patients who will/will not benefit from T-DM1. The same approach is being used to evaluate whether ^64^Cu-labeled trastuzumab can predict response to T-DM1 therapy (ClinicalTrials.gov identifier NCT02226276).

Mesothelin (MSLN) is highly expressed in pancreatic and ovarian cancer [169]. Molecular imaging with ^89^Zr-PET has been utilized in a phase I study to determine target antigen mesothelin expression, anti-mesothelin antibody tumor uptake, organ distribution at the whole body level, and the relationship between uptake and response to treatment in patients with advanced pancreatic and ovarian cancer. In this phase I study, ^89^Zr-PET imaging with anti-MSLN antibody MMOT0530A was performed before patients received antibody–drug conjugate DMOT4039A, containing MMOT0530A bound to cytotoxic payload MMAE [154]. Patients received 37 MBq ^89^Zr-MMOT0530A followed by PET/CT imaging 2, 4 and 7 days after injection. Maximum tumor uptake was detected four days post-injection. The SUV_max_ was 11.5 ± 7.5 in pancreatic cancer patients and 15.5 ± 8.7 in ovarian cancer patients. ^89^Zr-MMOT0530A uptake was observed in at least one tumor lesion in all patients (range 1–8). Six measurable lesions on diagnostic CT were not visible on PET scan. Within patients, a mean 2.4-fold difference in uptake between tumor lesions was observed. This difference was 5.3-fold between patients. Uptake in blood, liver, spleen and kidneys reflected normal antibody distribution. One patient out of eleven had a partial response, nine patients had stable disease and one patient had disease progression. Mean progression-free survival in this study was 121 days. Tracer tumor uptake was also correlated with immunohistochemistry of MSLN expression on archival tumor tissue. However, MSLN expression determined by IHC did not correlate with tracer uptake on PET scan in this study. This molecular imaging study with ^89^Zr-PET allowed visualisation and quantification of ADC uptake in pancreatic and ovarian cancer lesions. It also allowed the chance to study biodistribution at the whole body level [180].

These clinical studies highlight the importance of utilizing molecular imaging for ADC drug development, allowing evaluation of whole-body and tumor target expression. In order to guide ADC dosing in large clinical trials, these studies also provide vital information about in vivo ADC stability and the potential healthy organs at risk of toxicity.

## 8. Conclusions and Future Perspectives

ADC development is encouraged by the success of ADCs for treatment of various haematological malignancies and solid organ tumors. The growing ADC market is projected to reach US$4 billion by 2023 [5]. The successful development of ADCs requires a sophisticated understanding of target expression, ADC in vivo properties, drug delivery parameters and the pharmacodynamic profile of therapeutic activity in preclinical studies and in subsequent clinical trials. Combination therapies have the capability of reducing drug resistance and improving drug efficacy. Various clinical trials are now testing ADCs with other drugs including conventional chemotherapeutic regimens, tyrosine kinase inhibitors and checkpoint inhibitors in order to prevent the development of refractory disease. In future, ADC-based regimens would be incorporated earlier in the patient’s treatment course along with combination therapies to maximize therapeutic effects.

Molecular imaging has been successfully used in ADC development to study the biodistribution and pharmacodynamics of ADCs to determine target expression in the tumor and to detect interlesional heterogeneity in order to predict response to ADCs. As we use advanced molecular imaging methods to aid clinical decision making, we should consider several important issues. Firstly, the use of molecular imaging to guide clinical decision making requires prospective validation to demonstrate that initiating or ceasing treatment based on molecular imaging results in improved patient outcomes. Optimization of molecular imaging approaches and standardization of protocols will also assist in providing more accurate and reproducible data that can assist with decision making in drug development. In addition, advances in radiochemistry and conjugation approaches will result in improved immunoconjugate properties and facilitate the quantitative assessment of ADC target expression and drug delivery to the tumor in patients.

In conclusion, ADCs are drugs with enormous potential for the treatment of haematological malignancies and solid tumors, and strategies to manage toxicities and improve efficacy warrant further exploration. Advances in molecular imaging can make major contributions to ADC development in both preclinical studies and clinical trials, informing patient selection and optimizing outcomes.

## Figures and Tables

**Table 1:** Target antigens for ADCs in development and in in the clinic.

Indication	Targets
Acute myeloid leukaemia	CD25, CD33, CD123 (IL-3Rα), FLT3
Breast cancer	CD25, CD174, CD197 (CCR7), CD205 (Ly75), CD228 (P79, SEMF), c-MET, CRIPTO, ErbB2 (HER2), ErbB3 (HER3), FLOR1 (FRα), Globo H, GPNMB, IGF-1R, integrin β-6, PTK7 (CCK4), nectin-4 (PVRL4), ROR2, SLC39A6 (LIV1A ZIP6)
Bladder cancer	CD25, CD205(Ly75)
Colorectal cancer	CD74, CD174, CD166, CD227 (MUC-1), CD326 (Epcam), CEACAM5, CRIPTO, FAP, ED-B, ErbB3 (HER3)
Gastric cancer	CD25, CD197 (CCR7), CD228 (P79, SEMF), FLOR1(FRα), Globo H, GRP20, GCC, SLC39A6 (LIV1A ZIP6)
Gliomas GIII and GIV	CD25, EGFR
Head and neck cancer	CD71 (transferrin R), CD197 (CCR7), EGFR, SLC39A6 (LIV1A ZIP6)
Hodgkin’s lymphoma	CD25, CD30, CD197 (CCR7)
Lung cancer	Axl, alpha v beta6, CD25, CD56, CD71 (transferrin R), CD228 (P79, SEMF), CD326, CRIPTO, EGFR, ErbB3 (HER3), FAP, Globo H, GD2, IGF-1R, integrin β-6, mesothelin, PTK7 (CCK4), ROR2, SLC34A2 (NaPi2b), SLC39A6 (LIV1A ZIP6)
Liver cancer	CD276 (B7-H3), c-MET
Melanoma	CD276 (B7-H3), GD2, GPNMB, ED-B, PMEL 17, endothelin B receptor
Mesothelioma	Mesothelin, CD228 (P79, SEMF)
Multiple Myeloma	CD38, CD46 (MCP), CD56, CD74, CD138, CD269 (BCMA), endothelin B receptor
Non-Hodgkin Lymphoma	CD19, CD20, CD22, CD25, CD30, CD37, CD70, CD71 (transferrin R), CD72, CD79, CD180, CD205 (Ly75), ROR1
Ovarian cancer	CA125(MUC16), CD142 (TF), CD205 (Ly75), FLOR1(FRα), Globo H, mesothelin, PTK7 (CCK4)
Pancreatic cancer	CD25, CD71 (transferrin R), CD74, CD227 (MUC1), CD228 (P79, SEMF), GRP20, GCC, IGF-1R, integrin β-6, nectin-4 (PVRL4), SLC34A2 (NaPi2b), SLC44A4, alpha v beta6, mesothelin
Prostate cancer	CD46 (MCP), PSMA, STEAP-1, SLC44A4, TENB2
Renal cancer	AGS-16, EGFR, c-MET, CAIX, CD70, FLOR1 (FRα)

**Table 2:** Antibody–drug conjugates approved by the FDA for clinical use.

Payload	Target Antigen	Antibody–Drug Conjugates	Antibody	Drug to Antibody Ratio	Linker	Approved Indications	Year of FDA Approval	Year of EMA Approval
Calicheamicin derivative	CD22	Inotuzumab ozogamicin (Besponsa)	Recombinant humanized IgG4	5–7	Acid-labile hydrazone-based linker	B cell precursor ALL	2017	2017
	CD33	Gemtuzumab ozogamicin (Mylotarg)	Humanized IgG4	2–3	Acid-labile hydrazone-based linker	CD33-positive AML	2000 (withdrawn 2010); reapproved 2017	2018
DM1	ErbB2	Trastuzumab emtansine (T-DM1, Kadcyla),	Humanized IgG1	3.5	Non-cleavable thioether linker	ErbB2-positive metastatic breast cancer	2013	2013
MMAE	CD30	Brentuximab vedotin (SGN-35, Adcetris)	Chimeric IgG1	4	Protease-cleavable linker	Hodgkin’s lymphoma, ALCL, PTCL, mycosis fungoides	2011	2012
	CD79	Polatuzumab vedotin (Polivy)	Humanized IgG1	4	Protease-cleavable linker	DLBCL	2019	Not approved by the EMA
	Nectin-4	Enfortumab vedotin (ASG-22ME, Padcev)	Human IgG1	4	Cleavable valine-citrulline linker	Advanced urothelial cancer	2019	Not approved by the EMA
MMAF	BCMA	Belantamab mafodotin (GSK2857916, Blenrep)	Humanized IgG1	Unknown	Non-cleavable maleimidocaproyl (mc) linker	Relapsed/refractory multiple myeloma	2020	Orphan drug designation by the EMA, 2017
DXd (DX-8951 derivative)	ErbB2	Trastuzumab deruxtecan (DS-8201a, Enhertu)	Humanized IgG1	8	Cleavable peptide linker	Metastatic ErbB2-positive breast cancer	2019	Not approved by the EMA
SN-38	TROP2	Sacituzumab govitecan (IMMU-132, Trodelvy)	Humanized IgG1	7.6	Cleavable CL2A linker	Triple-negative breast cancer	2020	Not approved by the EMA
